# Similar Resilience Attributes in Lakes with Different Management Practices

**DOI:** 10.1371/journal.pone.0091881

**Published:** 2014-03-11

**Authors:** Didier L. Baho, Stina Drakare, Richard K. Johnson, Craig R. Allen, David G. Angeler

**Affiliations:** 1 Swedish University of Agricultural Sciences, Department of Aquatic Sciences and Assessment, Uppsala, Sweden; 2 U.S. Geological Survey, Nebraska Cooperative Fish and Wildlife Research Unit, School of Natural Resources, University of Nebraska – Lincoln, Lincoln, Nebraska, United States of America; Dauphin Island Sea Lab, United States of America

## Abstract

Liming has been used extensively in Scandinavia and elsewhere since the 1970s to counteract the negative effects of acidification. Communities in limed lakes usually return to acidified conditions once liming is discontinued, suggesting that liming is unlikely to shift acidified lakes to a state equivalent to pre-acidification conditions that requires no further management intervention. While this suggests a low resilience of limed lakes, attributes that confer resilience have not been assessed, limiting our understanding of the efficiency of costly management programs. In this study, we assessed community metrics (diversity, richness, evenness, biovolume), multivariate community structure and the relative resilience of phytoplankton in limed, acidified and circum-neutral lakes from 1997 to 2009, using multivariate time series modeling. We identified dominant temporal frequencies in the data, allowing us to track community change at distinct temporal scales. We assessed two attributes of relative resilience (cross-scale and within-scale structure) of the phytoplankton communities, based on the fluctuation frequency patterns identified. We also assessed species with stochastic temporal dynamics. Liming increased phytoplankton diversity and richness; however, multivariate community structure differed in limed relative to acidified and circum-neutral lakes. Cross-scale and within-scale attributes of resilience were similar across all lakes studied but the contribution of those species exhibiting stochastic dynamics was higher in the acidified and limed compared to circum-neutral lakes. From a resilience perspective, our results suggest that limed lakes comprise a particular condition of an acidified lake state. This explains why liming does not move acidified lakes out of a “degraded” basin of attraction. In addition, our study demonstrates the potential of time series modeling to assess the efficiency of restoration and management outcomes through quantification of the attributes contributing to resilience in ecosystems.

## Introduction

The capacity of an ecosystem to tolerate disturbances without changing its original structure, functions and processes has been defined as ecological resilience [1], [2]. Ecological systems can undergo regime shifts when disturbance thresholds are exceeded and reorganize in alternative states with new structures, functions and processes [3], [4]. Ecological consequences of regime shifts are uncertain, and sometimes they are considered to have negative consequences for biodiversity and ecosystem service provisioning to humans; for instance, when cultural eutrophication triggers a shift from a clear-water to a turbid-water state [4], [5]. After regime shifts, the new or alternative states can be stable, meaning that they resist returning to a state that existed prior to the regime shift [6]. In such cases, costly management and restoration interventions are needed to return ecosystems to resilient desired states [7], [8], [9], [10].

Clear examples of ecosystems that are trapped in a degraded state are acidified lakes [11], [12], [13], [14]. Although international agreements have resulted in reduced sulfur emissions to reduce acidification of freshwaters, several factors contribute to maintain lakes in an acidified state. Chemical weathering processes in soils [15], [16], biological interactions [17], biotic resistance [18], food web stability [19], limited dispersal and population connectivity [20], scale-specific processes [21], and Allee effects [12], [22] have been shown to constrain recovery to a desired, pre-acidification lake state. Thus, to protect sensitive biodiversity elements, especially the fish fauna, extensive liming programs have been established in many regions, including Sweden [23], [24], [25], [26], [27], [28]. Despite the consequences of liming on aquatic biota and ecosystem properties being increasingly understood [29], [30], it is unknown how liming affects the resilience of aquatic ecosystems.

Here, we describe an analysis of the resilience of phytoplankton community structure (resilience of what, [31]) in circum-neutral, acidified and limed lakes (i.e., lakes with different anthropogenic stress and management histories; resilience to what). We compare resilience characteristics of limed lakes relative to the degraded, undesired lake conditions (acidified lakes) and targeted reference conditions (circum-neutral lakes) (e.g. [12]), and more generally the efficiency of liming as a management tool. For such analyses, phytoplankton communities are appealing compared to other groups because they respond quickly to environmental change [6], [32] and are good indicators for tracking community changes in acidified [33], and limed lakes [29].

Theory and empirical evidence suggest that the dynamics of ecosystems are controlled by a small set of ecological processes that operate at distinct spatial and temporal scales [21], [34], [35]. The partitioning of structures and processes at multiple scales of time and space has important implications for the resilience of ecological systems [36], [37], [38], because resilience depends partly on how species, and the ecological functions they carry out, are distributed within and across scales [5], [39], [40]. It has been assumed that resilience increases with an increasing redundancy of species functions at a single scale, as well as how often these functions occur across scales [41]. Thus, a first step towards the empirical quantification of resilience is to make within and cross-scale structures explicit.

We use multivariate time series modeling, based on canonical ordination, to identify dominant temporal frequency fluctuation patterns in the phytoplankton communities [42]. Specifically, time series modeling identifies different temporal frequency patterns in the abundance or biomass structure of communities, which allows an assessment of the dynamic system structure that most likely arises from, and thus reflects, state-inherent system organization (e.g. feedbacks that characterize a basin of attraction). The method allows us to test for the presence of dominant temporal frequencies in species abundance or biomass, which in turn provides insight into temporal scaling patterns of communities [42], [43]. Enumerating the number of fluctuation frequencies or temporal scales present, allow us to quantify the cross-scale aspect of ecological resilience [44], [45]. We can then determine a second characteristic of resilience by examining the distribution of species within each of the temporal scales identified [44], [45]. Hypothetically, in a degraded ecosystem only a few dominant species might explain fluctuation frequencies at the temporal scales detected, whereas in a restored ecosystem both species richness within and the number of scales are higher. In this hypothetical example, the resilience of the restored system is deemed to be higher because of a higher within- and cross scale redundancy of patterns, suggesting a stronger reinforcement of processes and greater ability to buffer against disturbances.

Within and cross-scale structures in ecosystems are related to diversity [46]. To evaluate how diversity characteristics influence the within- and cross-scale structure of acidified, limed and circum-neutral lakes, we first evaluate metrics of community structure that are commonly used in ecology, followed by time series modeling. Based on our current ecological knowledge of limed and acidified lakes, we test the hypothesis that the relative resilience of limed lakes is lower relative to circum-neutral and acidified lakes. This lower resilience should manifest in a reduced within- and cross-scale structure arising from lime applications that we expect shall disrupt and homogenize natural community assembly processes. Results may provide a mechanistic basis for understanding why communities return to an acidified state once liming is discontinued [30].

## Materials and Methods

### Ethics Statement

All field sampling and laboratory analyses reported in this study are part of either the Swedish National Lake Monitoring Program or the national monitoring program for Integrated Studies of the Effects of Liming of Acidified Waters, both regulated by the Swedish Agency for Marine and Water Management. All data are made freely available to the public via the web by the data host (Department of Aquatic Sciences and Assessment; Swedish University of Agricultural Sciences; www.slu.se/aquatic-sciences) and no permission for use of the data is therefore required. It is also confirmed that the field studies did not involve endangered or protected species.

### Lake Selection

For this study, nine lakes representing three different management types were selected from the webpage hosting data from monitoring programs of Swedish inland waters (http://miljodata.slu.se/mvm/): 1) managed lakes (Ejgdesjön, Gyslättasjön, Gysltigesjön) that were limed to mitigate the effects of anthropogenic acidification; 2) acidified lakes (Brunnsjön, Härsvattnet, Rotehogstjärnen) that were unmanaged; 3) circum-neutral lakes (Allgjuttern, Fräcksjön, Stora Skärsjön), used as reference lakes with “desired” ecosystem properties. The study lakes are situated in the same ecoregion (i.e. the boreonemoral ecoregion of southern Sweden), are of similar size (mean lake surface area of 0.31 km^2^, range 0.11–0.83 km^2^) and have been monitored regularly for surface water chemistry and phytoplankton for 13 years (1997–2009).

### Sampling

Phytoplankton and two water chemistry variables that are directly related to liming (pH and calcium concentration) were sampled monthly during the ice-free period at a mid-lake station in each lake. Water was collected at 0.5 m depth with a Plexiglas® sampler and kept cool during transport to the laboratory for further analysis. All physicochemical analyses were done by SWEDAC certified laboratories (Swedish Board for Accreditation and Conformity Assessment, SWEDAC; http://www.swedac.se/en/) at the Department of Aquatic Sciences and Assessment, Swedish University of Agricultural Sciences, following International (ISO) or European (EU) standards when available [47]. A broader characterization of the abiotic environment of the lakes studied is shown in Electronic Table S1. Standard sampling protocols for abiotic and biological variables were used throughout the study period.

Phytoplankton was sampled by taking a water sample from the epilimnion using a 2-m long Plexiglas tube sampler (diameter = 3 cm). In lakes with a surface area >1 km^2^ a single mid-lake site was used for sampling. In lakes with a surface area <1 km^2^, five random epilimnetic water samples were taken and mixed to form a composite sample from which a subsample was taken and preserved with acid Lugol’s iodine solution [48]. Phytoplankton counts were made using an inverted light microscope and the modified Utermöhl technique commonly used in the Nordic countries [48]. Taxa were identified to the lowest taxonomic unit possible (usually species). Biovolumes (mm^3^ L^−1^) were calculated from geometric shapes following protocols developed by Blomqvist and Herlitz [49].

## Statistical Analyses

### Community and Water Quality Analysis

Prior to analyses, phytoplankton data were averaged to obtain three values that were spaced nearly equidistantly in time, covering early spring, summer and late autumn each year. We characterized phytoplankton community structure across lakes using common metrics (total biovolume, richness, diversity and evenness) following recent recommendations by Jost [50] and Tuomisto [51], [52] to obtain mathematically and statistically unbiased measures. The exponentiated Shannon index [50], which considers both species richness and evenness was used as a measure of “diversity” [51]. Exponentiation of the Shannon index expresses diversity in terms of species equivalents, making “diversity” and “richness” patterns directly comparable [50], [51]. Evenness was obtained by dividing “diversity” with “richness” and therefore unrelated to richness [50], [52].

Repeated measures analysis of variance (rm-ANOVA) was carried out in Statistica v.5 (Statsoft Inc, Tulsa, OK, USA) to test for significant differences in phytoplankton community metrics between the three categories of lakes. Similar rm-ANOVAs were conducted for pH and calcium concentration, to associate phytoplankton community dynamics with abiotic effects of liming. We tested for the effects of “management type” (circum-neutral, acidified and limed) (fixed factor), “time” (random factor) and their interactions. These factors comprised the independent variables while the community metrics comprised the dependent variables in the analysis. All data were log-transformed when necessary prior to analyses to fulfill the requirements of parametric tests. Because assumptions of sphericity were violated, degrees of freedom were corrected following the procedure by Huynh and Feldt [53] (note that this adjustment can lead to degrees of freedom with decimals). Inference was made at P<0.05. Tukey’s HSD test was performed to make a posteriori, group-wise comparisons of lake management types when a significant treatment effect was observed. We consider significant interaction terms between management type × time crucial for inferring differences in phytoplankton community metrics.

These univariate comparisons were complemented with multivariate analyses on phytoplankton communities using permutational multivariate analysis of variance in PERMANOVA version 1.6 [54]. PERMANOVA was based on a similar design as the univariate ANOVAs, using square root transformed species biovolume matrices that were converted in Bray–Curtis dissimilarity matrices and 9999 unrestricted permutations of raw data. Significant differences were inferred at an α-level of 0.05.

### Multivariate Time Series Modeling

We assessed two attributes of relative resilience (cross-scale and within-scale structure) of the phytoplankton communities with a time series modeling approach based on redundancy analysis (RDA) [42]. For an outline of the approach see the flow chart in Appendix S1 and Angeler *et al* [45]. We used temporal variables extracted by Asymmetric Eigenvector Maps (AEM) analysis [55], [56]. Briefly, the AEM analysis produces a set of orthogonal temporal variables that are derived from the linear time vector that comprises the length of the study period (i.e., 39 time steps for each lake) and that can be used as explanatory variables to model temporal relationships in community data. The type of AEM variables computed in the present study was designed for spatial analysis to account for linear trends in the response variables. As time comprises a directional process, AEM is better suited to model linear trends relative to other methods (Principal Coordinates of Neighbor Matrices and Moran Eigenvector Maps [56]). This procedure yielded AEM variables with positive Eigenvalues, each of which corresponds to a specific temporal structure and scale: the first AEM variable models linear trends and the subsequent variables capture temporal variability from slow to increasingly shorter fluctuation frequencies in the community data [57]. For each lake we constructed a parsimonious temporal model by running a forward selection on the AEM variables. Because AEM analysis is efficient in covering linear trends no detrending of models was necessary.

Redundancy analysis (RDA) retains significant AEM variables and these are linearly combined in ways to extract temporal patterns from the Hellinger-transformed species matrices (this transformation is achieved by dividing the species biovolumes by the row sum and taking the square root of the resulting values). The RDA identifies species with similar temporal patterns in the species × time matrix and uses their temporal pattern to calculate a modeled species group trend for these species based on linearly combined AEMs. The significance of the temporal patterns of all modeled fluctuation patterns of species groups revealed by the RDA is tested by means of permutation tests. The RDA relates each modeled temporal fluctuation pattern with a significant canonical axis. The R software generates linear combination (lc) score plots, which visually present the modeled temporal patterns of species groups that are associated with each canonical axis. Based on the number of significant canonical axes, the number of modeled fluctuation patterns of species groups with independent temporal patterns can be deduced. The ecological relevance of each temporal pattern identified can be quantified, using adjusted *R* ^2^ values of the canonical axes. The overall temporal structure of the whole community can then be deduced from the number of significant canonical axes in the RDA models. The number of canonical (RDA) axes identified gives insight about the number of temporal scales at which phytoplankton community fluctuations take place and can therefore be used to assess the cross-scale structure attribute of resilience [44], [45].

All relevant steps in the analyses were carried out with two functions implemented in the R 2.15.2 statistical software package [58]. First, the conversion of the linear time vector to AEM variables is done using the “aem.time” function (AEM package). All remaining steps (calculation of modeled species group trends, visual presentation of the results in form of lc score plots) are carried out with the “quickPCNM” function (PCNM package). All models are calculated exclusively based on an automatic statistical procedure, which limits bias in modeling scales that can be introduced by researcher subjectivity.

After identifying the cross-scale structure in phytoplankton community dynamics, we evaluated the within-scale attribute of resilience using correlation analysis. Spearman’s rank correlation analysis was used to investigate the relationship between individual phytoplankton species (raw biovolume data of individual species) with the linear combination (lc) scores extracted from significant canonical axes of the time series models for each lake type. With this approach we were able to calculate scale-specific taxon richness. Species that did not correlate with any canonical axes were considered to reflect stochastic dynamics. The number of these stochastic species was evaluated by subtracting the total number of species that correlated with canonical axes from the total number of species used for the time series modeling across lakes [45].

## Results

### Univariate and Multivariate Community Analyses

**Figure 1:**
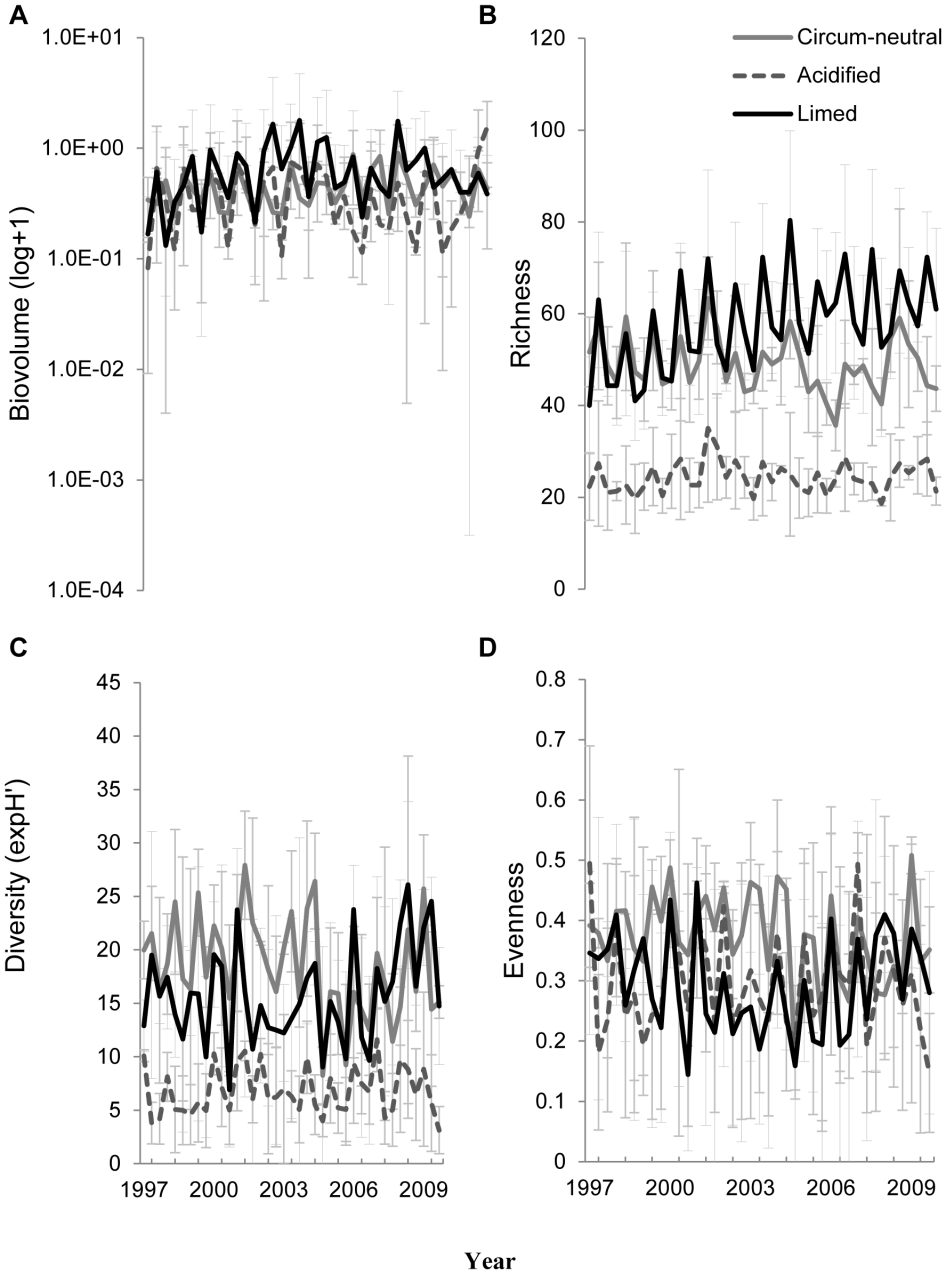
Comparison of the community metrics in the different lakes: (a) total biovolume, (b) species richness; (c) Shannon-Wiener diversity index; (d) evenness.

Univariate analyses of community metrics (Table 1; Figure 1) revealed significant lake management type effects for species richness and diversity but not for total biovolume and evenness. Post-hoc analysis revealed that the species richness and diversity were higher in circum-neutral lakes and limed lakes compared to acidified lakes (Tukey’s HSD test: circum-neutral = limed>acidified; P<0.05). The effect of time was significant for all metrics studied, but the interaction term (management type × time) was only significant for species richness. The results of the PERMANOVA identified a significant effect of management type and time whilst the interaction effect was not significant (Table 2). Groupwise analysis using PERMANOVA indicated that the phytoplankton community structure in the three different lake types were significantly different from each other (*P*<0.001).

**Table 1:** Results of repeated-measures ANOVA contrasting phytoplankton community metrics (total biovolume, richness, diversity and evenness) between lakes (circum-neutral, acidified and limed), time (year) and their interactions.

Metrics	Statistics	Treatment	Time	Treatment × Time
Total biovolume	d.f	3.71, 22.26	7.42, 22.26	7.42, 22.26
	MS	2.12	0.45	0.22
	F	0.15	2.01	0.98
	P	0.86	0.13	0.48
Richness	d.f	**10.36, 62.18**	**20.73, 62.18**	**20.73, 62.18**
	MS	**34973.35**	**262.93**	**100.42**
	F	**19.23**	**4.50**	**1.72**
	P	**<0.001**	**<0.001**	**0.05**
Diversity	d.f	**16.95, 101.70**	**33.90, 101.70**	33.90, 101.70
	MS	**4330.69**	**78.48**	32.07
	F	**12.27**	**2.53**	1.04
	P	**<0.001**	**<0.001**	0.43
Evenness	d.f	2, 6	**38, 228**	38, 228
	MS	0.24	**0.03**	0.01
	F	0.59	**3.08**	1.03
	P	0.584	**<0.001**	0.412

Significant terms are emphasized in bold.

(*df*: degrees of freedom Huynh-Feldt corrected, MS: mean squares, *F* ratio and *P* levels).

**Table 2:** Results of PERMANOVA contrasting phytoplankton communities between lakes (circum-neutral, acidified and limed), time (year) and their interactions.

Metrics	Statistics	Treatment	Time	Treatment × Time
Communities	d.f	**2, 6**	**38, 228**	76, 228
	MS	**8.28**	**0.27**	0.14
	F	**38.81**	**1.24**	0.65
	P	**<0.001**	**<0.001**	1

Significant terms are emphasized in bold.

(*df*: degrees of freedom, MS: mean squares, *F* ratio and *P* levels).

### Water Chemistry: pH and Calcium Concentration

**Figure 2:**
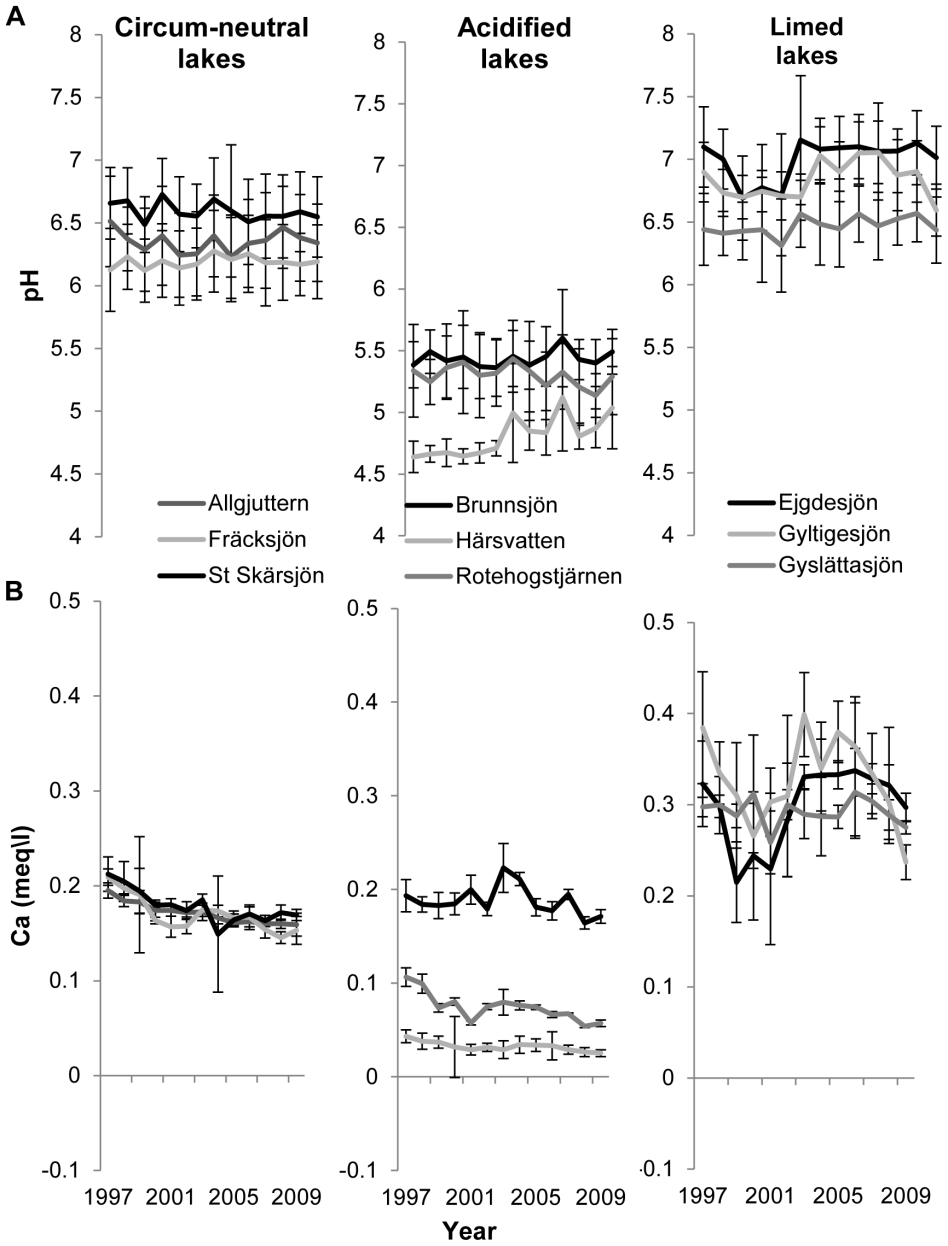
Selected water quality variables: (a) pH and (b) calcium concentration between 1997 and 2009 in circum-neutral acidified and limed lakes. Shown are the overall patterns (mean ± SE) for the different lakes.

The results of the repeated measures analysis of variance revealed that management type has an effect on both the pH and the calcium concentration, while the effect of time and the interaction term were not significant (Table 3 and Figure 2). Moreover, post-hoc analysis revealed that both the pH and the calcium concentration were higher in limed lakes than circum-neutral and acidified lakes, whereas circum-neutral lakes had higher values than acidified lakes (Tukey’s HSD test: Limed>circum-neutral>acidified; P<0.05).

**Table 3:** Results of repeated-measures ANOVA contrasting water quality variables (pH and calcium concentration) between lakes (circum-neutral, acidified and limed), time (year) and their interactions.

Parameters	Statistics	Treatment	Time	Treatment × Time
pH	d.f	**2, 6**	38, 228	76, 228
	MS	**80.25**	0.05	0.03
	F	**894.9**	0.56	0.31
	P	**<0.001**	0.98	1.00
Calcium (meq/L)	d.f	**2, 6**	38, 228	76, 228
	MS	**1.30**	<0.01	<0.01
	F	**463.07**	0.78	0.42
	P	**<0.001**	0.83	1.00

Significant terms are emphasized in bold.

(*df*: degrees of freedom, MS: mean squares, *F* ratio and *P* levels).

### Multivariate Time Series Modeling

**Figure 3:**
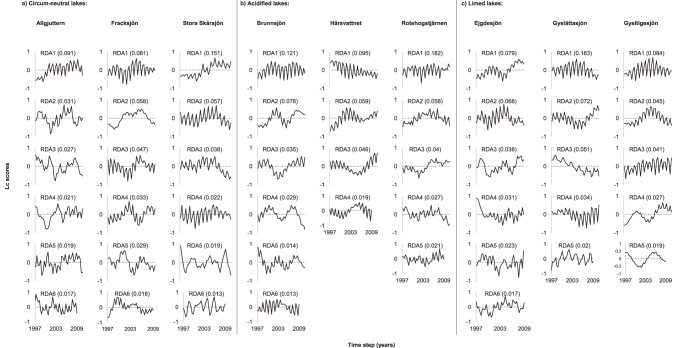
Lc score plot showing the temporal patterns of individual significant canonical with corresponding constrained variance for (a) circum-neutral lakes, (b) acidified lakes and (c) limed lakes.

Time series modeling revealed significant temporal structure in all lakes studied (Figure 3). The RDA models explained on average similar amounts of the adjusted variance of phytoplankton community dynamics (26% circum-neutral lakes, 27% acidified and 28% limed). The models show that the temporal fluctuation patterns of groups within the phytoplankton communities were unique in each lake independent of management practice. However, all circum-neutral lakes showed temporal dynamics at six significant temporal scales, compared to acidified (Brunnsjön 6, Härsvattnet 4, Rotehogstjärnen 5) and limed lakes (Ejgdesjön 6, Gyslättasjön 5, Gysltigesjön 5) (Figure 3), highlighting a slightly higher and more consistent cross-scale structure in circum-neutral lakes relative to the other lake types. Using Spearman rank correlation analysis (Table 4) to assess the within-scale attribute of resilience, we found that the number of species contributing to the scale-specific temporal patterns was comparable across the different lake types, suggesting that they have similar within-scale structures. Species that did not correlate with any significant temporal frequency pattern (i.e. stochastic species) were on average higher in limed (27%) and acidified (23%) compared to circum-neutral (15%) lakes.

**Table 4:** Spearman rank correlation analysis showing the number of species correlating with the significant canonical (RDA) axes from the multivariate time series analysis.

Lake	RDA1	RDA2	RDA3	RDA4	RDA5	RDA6	Stochastic	Total
Allgjuttern^a^	42(33)	19(15)	11(9)	11(9)	11(9)	14(11)	17(14)	125
Fräcksjön^a^	36(27)	17(13)	31(24)	14(11)	17(13)	7(5)	9(7)	131
Stora Skärsjön^a^	36(21)	20(11)	30(17)	19(11)	12(7)	13(7)	46(26)	176
Brunnsjön^b^	18(23)	15(19)	11(14)	2(3)	7(9)	7(9)	18(23)	78
Härsvatten^b^	15(29)	7(14)	8(16)	6(12)	–	–	15(29)	51
Rotehogstjärnen^b^	21(20)	20(19)	24(23)	13(14)	6(6)	–	19(18)	103
Ejgdesjön^c^	48(23)	38(19)	25(12)	16(8)	8(4)	14(7)	55(27)	204
Gyltigesjön^c^	37(13)	44(16)	46(16)	48(17)	10(4)	–	94(34)	279
Gyslättasjön^c^	54(20)	74(28)	40(15)	35(12)	13(5)	–	53(20)	269

The number of species that did not correlate with any significant axes was considered as “Stochastic” and the total number of species in the communities as “Total”. Values shown in parentheses are calculated percentages from the total.

a circum-neutral lakes,

b acidified lakes,

c limed lakes.

## Discussion

The relative resilience of limed lakes in terms of the dynamic within- and cross-scale structure of phytoplankton communities was predicted to be lower relative to acidified and circum-neutral lakes. The within-scale component of resilience, expressed as the percentage of species that explained each temporal pattern, was similar across lake types. Also the cross-scale structure differed marginally between managed and unmanaged lakes, although water chemistry accounted for subtle differences in cross-scale structure. All circum-neutral lakes and the least acidified Brunnsjön showed six distinct temporal frequency fluctuations, while the most acidified lake Härsvatten, that clearly comprises an undesired but stable state, had only four patterns. This pattern is counterintuitive because it suggests a lower resilience of this lake. This highlights that the cross-scale structure analysis, despite characterizing important features of complexity, may not capture the full spectrum of resilience. Our study shows how considering the dynamics of stochastic species can provide a broader picture of resilience. Similarly, despite liming substantially increasing pH and calcium concentration, no significant increase in cross-scale structure was observed, highlighting no pronounced effect of management action on phytoplankton cross-scale attributes of resilience. Although our study hypothesis was rejected, our results provided us with a better understanding how management action, specifically liming, influences resilience.

Management and restoration efforts are often directed towards breaking equilibrium conditions of undesired system states, returning them to more desired states and increasing the resilience of these restored or managed states [7], [8], [9], [10]. The finding of similar resilience characteristics across circum-neutral, acidified and limed lakes has several implications for management, especially regarding liming as a management tool [29], [59], [60]. The results can be interpreted in two contexts. (1) Circum-neutral lakes have been marginally affected by anthropogenic acidification because of their higher acid buffering capacity [61], [62]. The resilience characteristics observed in those lakes therefore comprises the target of management. The similar within - and cross-scale features of resilience observed in the acidified lakes suggest that natural recovery has putatively led to approaching targeted (circum-neutral lakes) resilience conditions. In this case, given the similar within- and cross scale resilience characteristics observed in limed lakes, we can conclude that costly management interventions do not necessarily achieve better conditions than unmanaged, natural recovery [60].

(2) The resilience characteristics observed in acidified lakes characterizes their basin of attraction and thus their resistance to return to desired target conditions [32]. This explanation seems more plausible, given that ecological recovery (i.e. resilience attributes and community composition) has not reached management targets in many cases despite decades of policy implementation [28], [63]. This interpretation is also supported by research that has identified many abiotic and biotic factors that constraint recovery [12], [13], [64]. How is this related to the resilience observed in limed lakes?

The similar within- and cross-scale attributes of resilience in limed and acidified lakes, suggest that liming produces at least a partial management success in terms of increasing species richness and diversity, which has also been observed in other studies [29], [65], [66]. However, from a systemic point of view, it seems to reorganize communities within the acidified states rather than break the feedbacks that maintain lakes in the acidified state. This re-organization, which was manifested in communities that are neither representative of acidified nor circum-neutral lakes, was evident in our PERMANOVA analysis (see also NMDS plot in Appendix S2) and the water chemistry analysis (see also [29]). From a resilience perspective, our results suggest that limed lakes comprise a particular condition of an acidified lake state; that is, liming keeps lakes in the “degraded” basin of attraction, thereby failing to restore and reinforce a desired state equivalent to pre-acidification conditions that maintains desired ecosystem attributes. Alternatively, if liming creates an alternative basin of attraction, it might be very shallow and therefore instable. Whether or not limed lakes comprise a particular configuration of an acidified state or an alternative state itself, our results support findings that have suggested that communities return to an acidified state once liming is discontinued [30]. Thus, the broader ecological implications of liming lakes are the following: 1) when natural recovery of acidified lakes attains similar resilience characteristics as in targeted reference lakes, costly management practice might not be necessary, 2) ecologists have begun to regard liming as an ecosystem-level perturbation rather than an integral restoration tool [67], [68], [69]. In a more specific management context, liming may comprise some form of command and control management [70], whereby the partial success in terms of targeted increase in diversity and species richness, and habitat suitability to sustain fisheries might generate substantial negative side effects in the ecosystem due to biogeochemical alteration [71]. If liming only partly mitigates acidification impacts without fundamentally altering the equilibrium conditions of acidified states (i.e. restoring acidified lakes to pre-acidification conditions that become self-sustaining), further research will be required to foster our understanding of potentially negative side effects on the ecological integrity of managed lakes (e.g. altered Al toxicity [72], altered nutrient precipitation [73], material consumption by invertebrates [74], food web structure [29]).

In addition to assessing patterns of resilience across managed and unmanaged boreal lakes, our study provides new insight into the consequences of liming for biodiversity and its effects on resilience. Increased resilience has been associated with a higher species richness and diversity in communities [5], [32]. While liming indeed increased the species richness and diversity in the limed lakes to targeted levels present in circum-neutral relative to acidified lakes, our time series models suggest that this increased diversity contributed little to within- and cross scale structure in the phytoplankton communities. This apparent paradox can be explained by the number of species with ostensibly stochastic dynamics that were on average higher in the limed and acidified compared to circum-neutral lakes. From a disturbance ecology perspective, our results are consistent with the findings of an increased importance of stochastic community assembly when ecosystems face perturbations [75], [76]; if this pattern can be generalized, the argument that liming comprises a perturbation would find additional support. This also suggests that the most acidified lake Härsvatten with the lowest cross-scale structure detected, might be able to cope with disturbances, thereby maintaining its functions and feedbacks, because of the higher amount of stochastic species increasing adaptive capacity (e.g. a high response diversity [77]). Our study makes clear how the role of species richness can be scrutinized if partitioned into patterns that reflect both the deterministic and stochastic processes occurring at different scales [45], thereby highlighting the usefulness of time series modeling for assessing resilience. These patterns can be further explored for gaining a more process-based understanding of how management affects species diversity and their influence on resilience.

We conclude by highlighting that, given the similar within- and cross-scale attributes observed across lakes, an assessment of resilience characteristic of limed lakes would have been inconclusive. Without information of the broader ecological impacts of liming on communities and other ecosystem characteristics that has accumulated in the literature [29], [30], [68], [69], [71], [72], [73], [74] and ecological knowledge of reference lakes, we would have not been able to judge whether lake liming achieved the ultimate management goal: of restoring and fostering desired ecosystem states. Assessments of the relative resilience of managed systems therefore require multiple lines of evidence, including reference sites that comprise management targets and approaches based on complex systems theory and “traditional” ways of characterizing community structure and functions to increase inference.

## Supporting Information

Table S1 Summary of geographical positions, morphological characteristics and water chemistry of study lakes. Values represent the inter-annual mean value and standard deviation for the study period 1997–2009.
(DOCX)

Appendix S1 **Flow chart outlining the steps involved in time series modeling.**
(DOCX)

Appendix S2 **Non-metric multidimensional scaling (NMDS) ordination showing phytoplankton communities across the different lake types over the study period (1997–2009).**
(DOCX)
