# Supplementary material for: Similar Resilience Attributes in Lakes with Different Management Practices
Source: PLoS One. 2014 Mar 11;9(3):e91881. doi: 10.1371/journal.pone.0091881 (PMC3950282; doi:10.1371/journal.pone.0091881)
Supplement: Table S1 — Summary of geographical positions, morphological characteristics and water chemistry of study lakes. Values represent the inter-annual mean value and standard deviation for the study period 1997–2009. (DOCX) [file pone.0091881.s001.docx]

Electronic Table S1.

Baho *et al.* (2014): Similar resilience attributes in lakes with different management practices

Summarizing geographical position, morphological characteristics and water chemistry of study lakes. Values represent the inter-annual mean value and standard deviation for the study period 1997-2009.

| **Lake** | **Coordinates** | | **Lake size** (km^2^) | **Water temp.** (°C) | **pH** | **Electr. Cond.** (mS cm^-1^) | **Alkalinity**  (meq L^-1^) | **SO_4_**  (meq L-1) | **PO4-P** (µg/L) | **Total P** (µg L-1) | **Total N** (µg L-1) | **TOC** (mg L-1) |
| --- | --- | --- | --- | --- | --- | --- | --- | --- | --- | --- | --- | --- |
|  | X_SMHI | Y_SMHI |  |  |  |  |  |  |  |  |  |  |
| *Circum-neutral* |  |  |  |  |  |  |  |  |  |  |  |  |
| Allgjuttern | 642489 | 151724 | 0.19 | 8.65 ± 6.08 | 6.35 ± 0.34 | 4.67 ± 0.40 | 0.07 ±  0.12 | 0.17 ±  0.02 | 1.96 ± 1.02 | 6.82 ± 3.51 | 369.34 ± 84.01 | 7.08 ± 0.78 |
| Fräcksjön | 645289 | 128665 | 0.28 | 9.35 ± 5.63 | 6.18 ± 0.29 | 6.39 ± 0.62 | 0.07 ±  0.02 | 0.13 ±  0.04 | 2.73 ± 1.50 | 9.93 ± 4.73 | 489.51 ± 125.44 | 9.53 ± 1.38 |
| Stora Skärsjön | 628606 | 133205 | 0.31 | 10.43 ± 5.32 | 6.60 ± 0.32 | 7.74 ± 0.86 | 0.13 ±  0.05 | 0.17 ±  0.03 | 3.09 ± 2.11 | 10.01 ± 5.12 | 484.72± 208.85 | 4.74 ± 1.50 |
| *Acidified* |  |  |  |  |  |  |  |  |  |  |  |  |
| Brunnsjön | 627443 | 149526 | 0.11 | 7.68 ± 5.61 | 5.42 ± 0.23 | 6.03 ± 0.63 | 0.004 ±  0.01 | 0.199 ± 0.04 | 5.16 ± 3.61 | 15.40 ± 11.85 | 722.78 ± 195.09 | 19.98 ± 4.78 |
| Härsvatten | 643914 | 127698 | 0.19 | 9.91 ± 6.41 | 4.80 ± 0.25 | 5.42 ± 0.74 | -0.03 ±  0.01 | 0.11 ±  0.03 | 1.84 ± 1.62 | 7.54 ± 9.56 | 396.98 ± 191.00 | 3.82 ± 2.49 |
| Rotehogstjärnen | 652902 | 125783 | 0.17 | 9.00 ± 4.94 | 5.30 ± 0.30 | 4.71 ± 0.78 | -0.001±  0.02 | 0.10 ±  0.04 | 3.20 ± 1.65 | 16.76 ± 9.85 | 455.75 ± 113.89 | 12.87 ± 2.56 |
| *Limed* |  |  |  |  |  |  |  |  |  |  |  |  |
| Ejgdesjön | 653737 | 125017 | 0.83 | 9.77 ± 6.11 | 7.00 ± 0.34 | 7.38 ± 0.97 | 0.22 ±  0.07 | 0.08 ±  0.02 | 1.24 ± 0.61 | 4.78 ± 3.20 | 430.92 ± 89.11 | 5.72 ± 1.08 |
| Gyltigesjön | 629489 | 133906 | 0.4 | 9.34 ± 5.55 | 6.84 ± 0.25 | 7.11 ± 1.10 | 0.20 ±  0.07 | 0.11 ±  0.03 | 4.42 ± 1.90 | 15.30 ± 6.54 | 627.12 ± 127.98 | 11.98 ± 4.20 |
| Gyslättasjön | 633209 | 141991 | 0.33 | 9.51 ± 5.12 | 6.74 ± 0.28 | 6.09 ± 0.96 | 0.12 ±  0.04 | 0.13 ±  0.03 | 2.95 ± 1.77 | 14.95 ± 6.35 | 538.71 ± 167.36 | 12.23 ± 1.70 |
