# Supplementary material for: Similar Resilience Attributes in Lakes with Different Management Practices
Source: PLoS One. 2014 Mar 11;9(3):e91881. doi: 10.1371/journal.pone.0091881 (PMC3950282; doi:10.1371/journal.pone.0091881)
Supplement: Appendix S1 — Flow chart outlining the steps involved in time series modeling. (DOCX) [file pone.0091881.s002.docx]

*Appendix S1*

# Baho *et al.* (2014): Similar resilience attributes in lakes with different management practices

Flow chart outlining the steps involved in time series modeling.


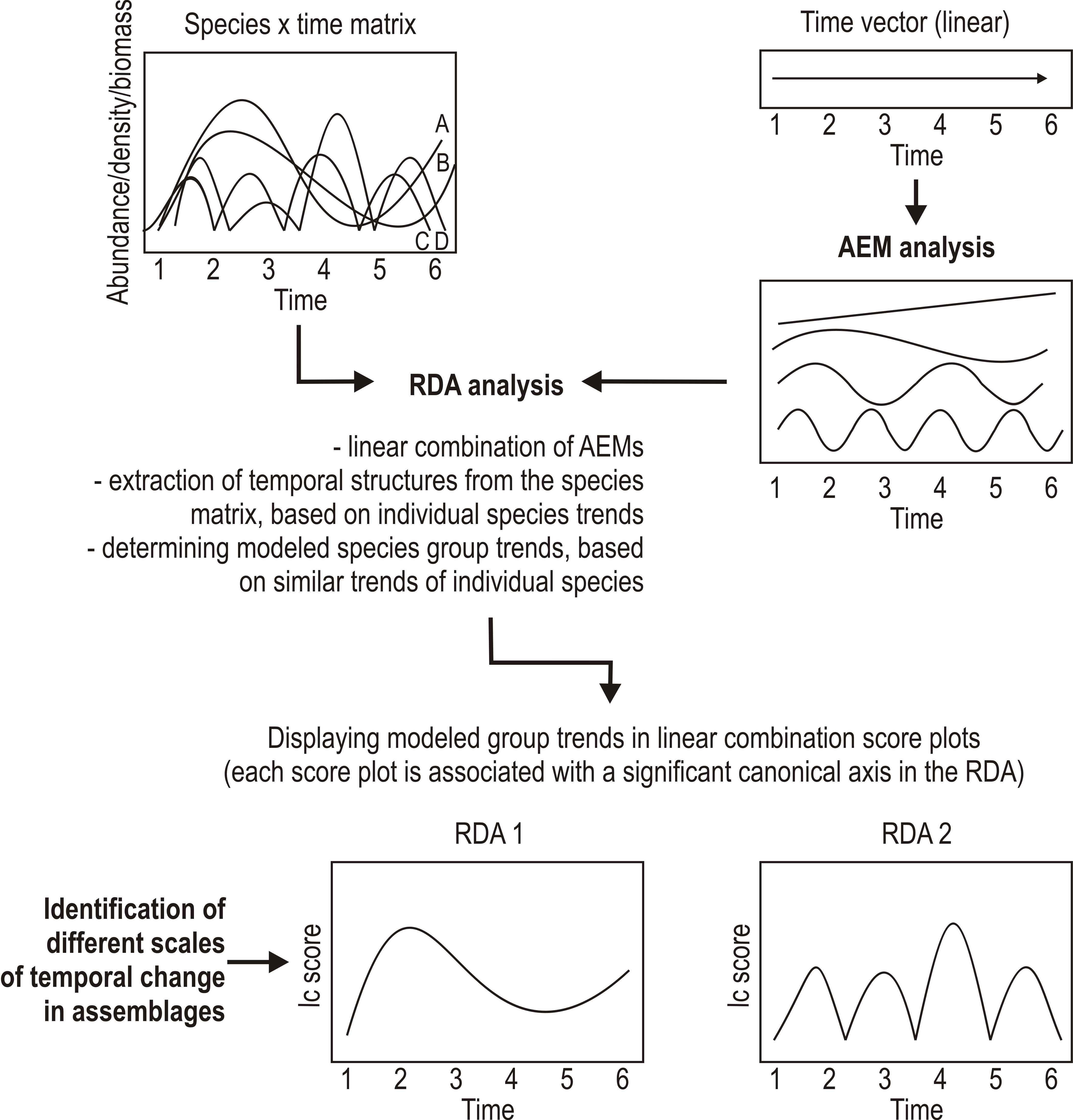


First, a linear time vector is converted into AEM (Asymmetric Eigenvector Maps) variables, which are then related to the species 🞨 time matrix of a specific ecosystem by means of RDA. The RDA identifies species with similar temporal trends in the species 🞨 time matrix and calculates a modeled species group trend from their collective patterns. Significant modeled trends are associated with significant RDA axes, and these trends are visually shown in linear combination score plots. Because RDA axes are independent from each other, they represent temporal patterns at independent scales.
