# Supplementary material for: Similar Resilience Attributes in Lakes with Different Management Practices
Source: PLoS One. 2014 Mar 11;9(3):e91881. doi: 10.1371/journal.pone.0091881 (PMC3950282; doi:10.1371/journal.pone.0091881)
Supplement: Appendix S2 — Non-metric multidimensional scaling (NMDS) ordination showing phytoplankton communities across the different lake types over the study period (1997–2009). (DOCX) [file pone.0091881.s003.docx]

*Appendix S2*

# Baho *et al.* (2014): Similar resilience attributes in lakes with different management practices

Non-metric multidimensional scaling (NMDS) ordination showing phytoplankton communities across the different lake types for the study period (1997-2009).

Stress: 0.12
